# Enhanced surface activation of ground tire rubber via the radiolysis of water for effective rubber recycling

**DOI:** 10.1016/j.heliyon.2024.e37454

**Published:** 2024-09-05

**Authors:** Lóránt Kiss, Alexandra Erzsébet Berényi, Miklós Németh, Anna Tegze, Renáta Homlok, Erzsébet Takács, László Mészáros

**Affiliations:** a Department of Polymer Engineering, Faculty of Mechanical Engineering, Budapest University of Technology and Economics, Műegyetem rkp. 3., H-1111 Budapest, Hungary; b Department of Surface Chemistry and Catalysis, Institute for Energy Security and Environmental Safety, HUN-REN Centre for Energy Research, Konkoly-Thege M. street 29-33, H-1121 Budapest, Hungary; c Radiation Chemistry Department, Institute for Energy Security and Environmental Safety, HUN-REN Centre for Energy Research, Konkoly-Thege M. street 29-33, H-1121 Budapest, Hungary; d HUN-REN-BME Research Group for Composite Science and Technology, Műegyetem rkp. 3., H-1111 Budapest, Hungary

**Keywords:** Polymer waste, Rubber recycling, Gamma radiation, Surface characteristics, Water radiolysis, Surface activation

## Abstract

In this study, we examined the chemical changes occurring in ground tire rubber (GTR) and on its surface as a result of gamma irradiation in water, with low doses of 5, 10, 15, 20, 25, and 30 kGy. To better distinguish the changes the radiation caused in the GTR and the surface activation of the GTR caused by the irradiated water, we also performed radiation treatments in an inert atmosphere. We mixed the treated GTRs with fresh rubber, and after vulcanization, investigated the mechanical properties and conducted dose optimization. The chemical changes occurring in GTR were characterized by Soxhlet-extraction and cross-link density measurements. Changes on the surface were investigated by Fourier transform infrared spectroscopy (FTIR) and X-ray photoelectron spectroscopy (XPS). In water irradiation, cross-link density decreased (∼10 %), while in an inert atmosphere, new bonds formed between the chains (∼10 %), with negligible degradation (∼2 %) in both cases. Based on the FTIR spectra, new oxygen-containing groups appeared on the GTR surface in water treatment, while this was not observed in a nitrogen atmosphere. The increase in surface oxygen content was confirmed by XPS, showing a ∼10 % increase as a result of water treatment, while it remained unchanged in the inert atmosphere. We achieved a 30 % increase in tensile strength (5 kGy) without a decrease in elongation properties and a 32 % increase in tear strength (20 kGy) for vulcanizates containing surface-activated GTR. Mechanical properties did not improve with treatments in an inert atmosphere. The oxidizing agents formed during the radiolysis of water activated the surface of the GTR, helping to establish a better connection with the matrix.

## 1 Introduction

Elastomers are utilized in seals and vibration-damping components, but tire manufacturing constitutes the biggest portion of the global rubber industry [1,2]. In 2020, over 324 million new tires were sold in Europe alone, contributing to a substantial amount of waste generation. The amount of waste tires is projected to increase to 5 billion by 2030 [3,4]. Although tire collection rates have increased to nearly 95 % in Europe, only 42 % are recycled [4]. The environmental risks of tires ending up in landfills include soil contamination and the threat of unquenchable tire fires, releasing carcinogenic substances [5]. In order to reduce the environmental impact and contribute to a circular economy, it is necessary to increase the recycling rate, for which the development of new recycling technologies is essential.

Currently, the most common method of recycling end-of-life tires is based on grinding, followed by using the resulting ground tire rubber (GTR) in new matrices [1,3,4,6,7], with fresh rubber [8,9]. However, in many cases, the GTR cannot effectively work together with the matrix materials, limiting the applicability of the final products [1,[10], [11], [12], [13]]. Several approaches have emerged to improve interphase compatibility, such as devulcanization [[14], [15], [16], [17]], where the goal is to selectively cleave the cross-links in the GTR. Zhang et al. [18] a mechanochemical devulcanization process and then utilized the GTRs in natural rubber–based vulcanizates. Tensile strength, elongation at break, and tear strength significantly increased due to the treatment, with more pronounced effects at lower GTR content. Simon and Bárány [14] used microwave devulcanization, also followed by incorporating the GTR into natural rubber–based vulcanizates. The treatments led to a substantial decrease in cross-link density, although it was insufficient to improve the mechanical properties of the mixtures. However, the two-step mixing of a mixture containing devulcanized GTR led to increased tensile strength and tear strength.

Another possible method to improve compatibility between phases is modifying the surface of GTR [19]. Active groups are introduced onto the surface (*e.g.*, oxidation), usually through chemical treatments [[19], [20], [21]] and occasionally through radiation technologies [[22], [23], [24]], to facilitate better interaction with the matrix. In this case, exploring the changes on the surface is crucial but often challenging. Cao et al. [25] utilized ozone for oxidizing the surface of the ground rubber and examined the resulting changes using FTIR and XPS. They demonstrated that the treatment led to the appearance of new peaks associated with carbonyl and hydroxyl groups in the FTIR spectrum, and the quantity of surface oxygen increased by approximately 3 %. He et al. [26] chemically (KMnO_4_) treated the surface of GTR by oxidation, and subsequently confirmed the appearance of new carbonyl groups. The treated ground rubber was applied in a cement matrix, resulting in improved mechanical properties due to the treatment.

In recent years, the applicability of radiation technologies for polymer waste has become an intensively researched area and includes cross-linked systems (*e.g.*, GTR) as well [27]. Ionizing radiation treatments are often applied after mixing [[28], [29], [30]], but using them as a surface activation treatment has several advantages. Sonnier et al. [22] treated GTR with gamma radiation in air and determined the presence of oxygen-containing groups on the surface, based on the FTIR spectrum. Similarly, Ratnam et al. [31] treated GTR with gamma radiation in an air atmosphere and then used the ground rubber in an ethylene-vinyl acetate (EVA) matrix. As a result of surface activation, both tensile strength and elongation at break increased, indicating improved interaction between the phases. Unfortunately, the application of radiation treatments in vulcanizates containing rubber powder is a less-explored area but based on our preliminary studies [32,33], it appears to be a promising field.

In a previous study [33], we demonstrated that the surface activation of GTR can be performed through ionizing radiation treatment in an aqueous environment with continuous air bubbling. Surface activation occurs due to the strong oxidizing agents (primarily OH∙, H_2_O_2_) generated during the process. When the treated GTR is applied in a fresh rubber matrix, its mechanical properties (tensile strength, tear strength, etc.) improve significantly. The best results were obtained with a dose of 20 kGy, but experiments were not conducted with lower doses. However, reducing the required dose is crucial for future industrial implementation, and our goal in this study is to determine an optimal low dose. It is also important to distinguish the changes in the chemical structure and on the surface due to radiation, which provides deeper insights into the improved compatibility between the GTR and the matrix. This aspect has not been addressed in the literature, so in this study, we examined GTRs irradiated in an inert atmosphere and in a water environment separately. This allows us to isolate these effects, as surface activation does not occur in an inert environment due to the absence of oxygen.

Therefore, in this study, we treated GTR using gamma radiation in a water medium and a nitrogen atmosphere with low doses (<30 kGy). In order to examine the chemical changes that occurred in the GTR, we conducted cross-link density and Soxhlet extraction tests. To investigate the chemical changes on the surface, we characterized them using FTIR and XPS spectroscopy. Furthermore, we examined the applicability of the treated GTRs in natural rubber-based blends. We comprehensively analyzed the mechanical, vulcanization, and morphological properties of the vulcanizates containing GTR. We performed the experiments on irradiated GTR in an inert (nitrogen) atmosphere, using identical doses, to separate the effects of surface activation and radiation-induced chemical changes (chain scission, cross-link formation) occurring in the GTR.

## 2 Materials and methods

### 2.1 Materials

Ground tire rubber was provided by Aquajet Ltd. (Budapest, Hungary); it was obtained from the side wall and tread of truck tires with the use of water-jet milling. The particle size distribution can be found in Table 1. Detailed information (composition, morphology) about the used GTR can be found in our previous publication [32].

**Table 1:** Particle size distribution of the applied ground tire rubber.

Size (μm)	Mass percentage (%)
>500	1.7
250–500	21.7
125–250	55.9
75–125	16.1
<75	4.6

We chose a general-purpose natural rubber (NR), CV 60, for our experiments. The rubber mixtures contained the following additives: stearic acid (Oleon, Ertvelde, Belgium), zinc oxide (Werco Metal, Zlatna, Romania), paraffin oil (Hansen und Rosenthal, Hamburg, Germany), N-cyclohexyl-2-benzothiazolesulfenamide (CBS, Rhein Chemie, Mannaheim, Germany), tetramethylthiuram disulfide (TMTD, Lanxess, Cologne, Germany), N772 carbon black (Omsk Carbon Group, Omsk, Russia) and sulfur (Ningbo Actmix Polymer, Ningbo, China).

### 2.2 Radiation treatment of ground tire rubber

The GTR was subjected to radiation treatment at the Institute of Isotopes Ltd. (Budapest, Hungary). Radiation was provided by a panoramic SLL-01 ^60^Co source. A constant dose rate of 2 kGy/h was maintained in all cases, and the absorbed doses were as follows: 5, 10, 15, 20, 25, and 30 kGy. To prepare the samples irradiated in water, we placed ground tire rubber (100 g) in sealable containers, which we filled with distilled water (400 ml). In order to reduce surface tension and facilitate suspension, we added 2 ml of Triton X-100 (Sigma-Aldrich, Missouri, USA) surface active material (non-ionic surfactant) to the suspension. This step was necessary as the hydrophobic nature of GTR would have caused it to float on the surface of the water otherwise. Air was continuously bubbled into the containers through a silicone tube to ensure the presence of oxidizing agents produced during radiolysis. It also ensured that the GTR did not settle at the bottom of the container. Following the treatment, the water-based suspensions were passed through pleated filter paper (615 MN). Subsequently, we left them to dry naturally at room temperature until they reached a constant mass.

**Fig. 1:**
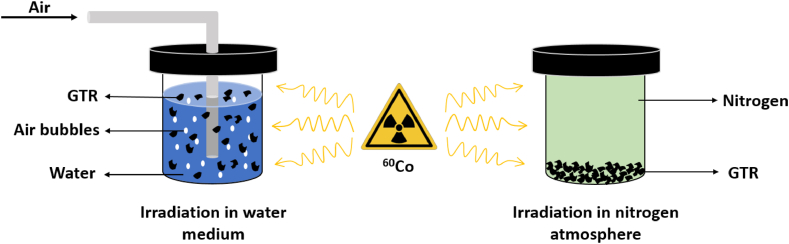
Arrangements used during ionizing radiation treatments.

During the radiation treatment under an inert atmosphere, the GTR was rinsed with nitrogen in a sealable glass container, which was then tightly closed. The containers were airtight, preventing the entry of oxygen during the treatment. The radiation parameters were the same as in the water medium–based method. The arrangements used for both radiation treatments can be seen in Fig. 1.

### 2.3 Characterization of ground tire rubber

In order to explore the chemical changes in the GTR caused by radiation, we determined the sol fractions of the GTRs by conducting Soxhlet extractions as per Eq. (1). Toluene served as the solvent, and the extractions were run for 16 h. Subsequently, the samples were dried until they reached a constant weight at 80 °C. We weighed the samples twice: prior to extraction and after drying. We calculated the sol fractions based on Eq. (1).

(1) $$Sol\;fraction\;\left(\%\right)=\left(1-\frac{m_{f}}{m_{i}}\right)∙100\text{,}$$

where *m*_*i*_ and *m*_*f*_ stand for the mass of GTRs before and after the extraction, respectively.

The cross-link densities of the ground tire rubbers were measured with swelling tests following the ASTM D 6814-02 standard and with the use of the Flory-Rehner equation (Eq. 2.) [34]. The tests were performed in toluene for a duration of 72 h, then the samples were dried at 80 °C until they reached a constant mass.

(2) $$\nu_{e}=\frac{-\left[\ln\left(1-V_{r}\right)+V_{r}+\chi ∙{V_{r}}^{2}\right]}{\left[V_{s}∙\left({V_{r}}^{\frac{1}{3}}-\frac{V_{r}}{2}\right)\right]}\text{,}$$

where $\nu_{e}$ is cross-link density (mol/dm^3^), *V*_*s*_ is the molar volume of the solvent (in the case of toluene: 0.10627 dm^3^/mol), χ is the rubber–solvent interaction parameter (0.39), and *V*_*r*_ is the volume fraction of the rubber in the swollen sample, which can be calculated with the Ellis and Welding [35] equation (Eq. 3.).

(3) $$V_{r}=\frac{\frac{m_{r}}{\rho_{r}}}{\frac{m_{r}}{\rho_{r}}+\frac{m_{s}}{\rho_{s}}}\text{,}$$

where *m*_*r*_ is the mass of the dry rubber (g), *m*_*s*_ is the weight of the solvent absorbed by the sample (g), *ρ*_*s*_ is the density of the solvent (866.9 g/dm^3^), and *ρ*_*r*_ is the density of the GTR (1200 g/dm^3^).

The surface changes were examined with a Bruker Tensor II (Bruker Corporation, Billerica, USA) Fourier transform infrared spectroscope in attenuated total reflection (ATR) mode. The measurements were conducted in the wavelength range of 400–4000 cm^−1^ with a resolution of 4 cm^−1^. During the evaluations, the spectrum of the untreated sample (reference) was subtracted from the spectra of the radiation-treated GTR samples, thereby enhancing the visible differences and facilitating the assessment of surface changes.

In order to determine the composition and chemical state of the GTR surfaces, we performed X-ray photoelectron spectroscopy (XPS) in a Thermo Scientific ESCALAB Xi^+^ (Thermo Fischer Scientific, Waltham, USA) instrument. A monochromatized Al K-alpha source (1486.6 eV) was used with a 900 μm spot size. At least 5 different spots were analyzed on all samples. On each sample, we collected wide-range spectra (at a pass energy of 80 eV) to analyze elemental composition. We recorded high-resolution spectra (at 20 eV pass energy) for the following photoelectron lines: C 1s, O 1s, and Zn 2p for quantitative and chemical state analysis. The charging of the surface of the sample was compensated for by the instrument's automatic built-in dual charge compensation system. We used the C-C/C-H component of the C 1s peak as a reference (284.8 eV) to slightly adjust the energy scale.

### 2.4 The production of vulcanizates

In this study, we used so-called two-step mixing to prepare the NR-based vulcanizates containing GTR. Our previous studies [32,33] show that with this technology, we can produce vulcanizates with better mechanical properties using surface-activated GTR compared to the commonly used single-step mixing, where each component is added in a single mixing step. The essence of two-step mixing is that the GTR is not added to the mixture directly. Instead, it is blended with a vulcanizing system (GTR pre-mixture) in a preceding separate step. Then, in the 2nd step, this pre-mixture is introduced into the matrix along with the other components. Based on these, we prepared the GTR pre-mixtures for every dose and atmosphere (Table 2). The order of materials in the table represents the sequence of mixing from top to bottom.

**Table 2:** GTR pre-mixture recipe (GTR* stands for the appropriate atmosphere and absorbed dose).

Component	Amount (phr)
ZnO	5
Stearic acid	2
GTR*	100
Paraffin oil	10
CBS	1.25
TMTD	0.6
Sulfur	0.6

Subsequently, in the second step, using the previously produced pre-mixtures, we prepared the final rubber mixtures, the composition of which is available in Table 3. The order of materials represents the sequence of mixing from top to bottom. The mixtures were prepared in a Brabender Lab-Station internal mixer (Brabender GmbH & Co. KG, Duisburg, Germany) at a temperature of 50 °C and a rotor speed of 40 rpm.

**Table 3:** Rubber mixture recipe (GTR*_premix stands for the treated GTR).

Component	Amount (phr)
NR	100
CB	60
ZnO	5
Stearic acid	2
GTR*_premix	100
Paraffin oil	10
CBS	1.25
TMTD	0.6
Sulfur	0.6

To determine the curing characteristics of the compounds, we captured the vulcanization curves using a MonTech D-RPA 3000 moving die rheometer (MonTech Werkstofprüfmaschinen GmbH, Buchen, Germany). The tests were performed at 160 °C with an amplitude of 1° and a frequency of 1.67 Hz for a duration of 20 min. We determined the vulcanization times (t_90_), maximum torque (S'_max_), and the peak rates.

The compounds were vulcanized with a Teach-Line Platen Press 200E hydraulic press (Dr. Collin GmbH, Munich, Germany) at a temperature of 160 °C and a pressure of 2.8 MPa. Each compound was compressed into sheets measuring 200 mm × 200 mm x 2 mm, and the curing process continued until t_90_.

### 2.5 Testing of vulcanizates

The mechanical properties of the vulcanizates were determined with a Zwick-Z005 universal testing machine (Zwick GmbH., Ulm, Germany) at room temperature. We conducted tests following the DIN 53504 standard to determine the tensile properties of the vulcanizates. Type 1 specimens were used with a clamping distance of 60 mm and a crosshead speed of 500 mm/min. Tear strength tests were performed according to the ASTM D624 standard, with type C specimens with a test speed of 500 mm/min and a clamping distance of 56 mm.

The morphology of the fracture surfaces of the tear test specimens was examined through scanning electron microscopy. Scanning electron micrographs were captured with a JEOL JSM 6380LA scanning electron microscope (Jeol Ltd., Tokyo, Japan) after the specimens were sputtered with gold.

## 3 Results and discussion

### 3.1 Properties of the ground tire rubber

**Fig. 2:**
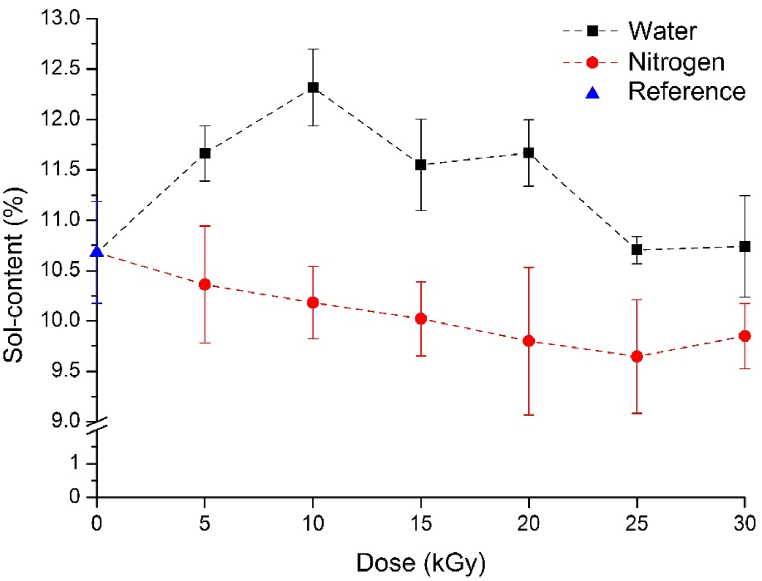
Sol-content of the irradiated GTRs in nitrogen and water.

The results of the Soxhlet extractions are shown in Fig. 2. The results indicate that, compared to the reference, soluble material content decreased as a result of irradiation in an inert atmosphere, while it increased in a water medium. In a nitrogen atmosphere, mild cross-linking likely occurred, a characteristic behavior of polymers when irradiated in an oxygen-free environment. In the water medium, the strong oxidizing agents cleaved small molecular fragments from the chain, thus increasing the soluble material content. However, even with 30 kGy, this increase is approximately ∼2 %, which is minimal. It is advantageous because if extensive degradation had occurred, it would have impaired the mechanical properties of the vulcanizates [32]. In the case of small doses (0–30 kGy), the medium used during radiation caused significant differences in the reactions taking place. However, these changes were small—they had a negligible impact on the vulcanizates.

**Fig. 3:**
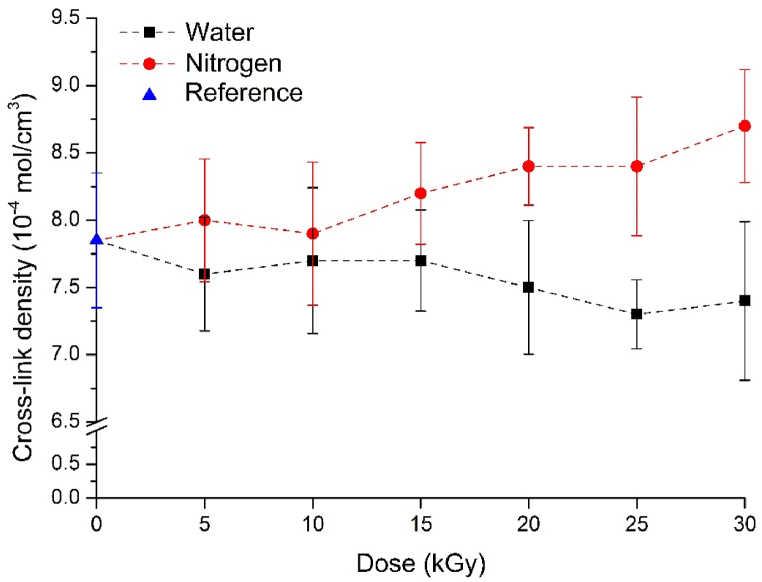
Cross-link density of the irradiated GTRs in nitrogen and water.

Cross-link density is shown in Fig. 3. In the case of GTRs irradiated in a water medium, cross-link density slightly decreased compared to the reference (untreated), but even with the greatest reduction (at 25 kGy) was under 10 %. Due to the high energy of gamma radiation, the bonds may break, and also, the oxidizing agents formed during radiolysis can attack cross-links, thereby reducing cross-link density. In the case of GTRs treated in an inert atmosphere, we observed an opposite process. Cross-link density increased compared to the reference, and the largest increase (at 30 kGy) was approximately 10 %. The high energy of the radiation causes radicals to form on the chains (*e.g.* breaking of double bonds, detachment of hydrogen atoms), which can recombine with radicals on other polymer chains, thereby creating new bonds between backbones. The differences in cross-link density also confirmed that significantly different reactions occurred due to the influence of different media during the radiation treatment. However, the change in cross-link density is very small in the examined range, so it will not have a significant impact on the properties of vulcanizates in later applications with fresh rubber.

**Fig. 4:**
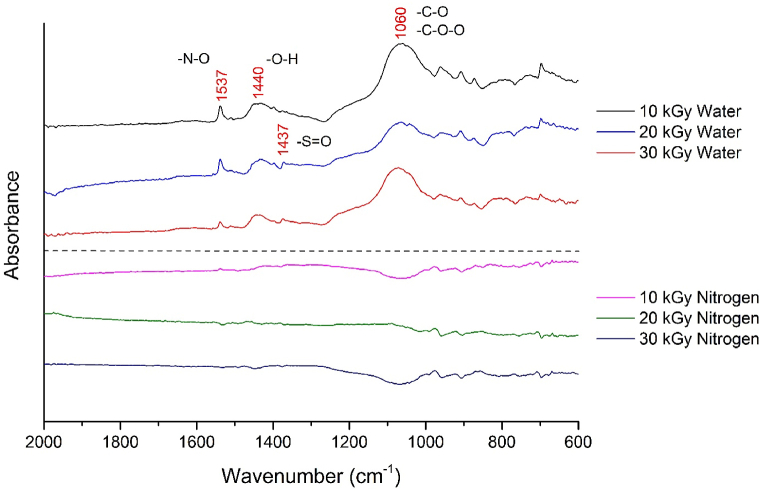
The difference in the FTIR spectra of irradiated GTR samples (the spectrum of the untreated GTR was subtracted from the measured spectra).

Fig. 4 shows the difference in the FTIR spectra of the radiation treated GTRs in water medium and nitrogen atmosphere (the dotted line divides the results of the two different treatments). In order to examine the chemical changes that occurred on the surface, we used the spectrum of the reference (untreated GTR) as a background, making the changes induced by radiation more visible. Due to the nature of FTIR-ATR analysis, we can only obtain information about the presence of groups, not their quantity. The spectra primarily show N-O groups, hydroxyl groups (O-H), and sulfonate groups (S=O) [36]. Another significant peak is visible in the range of roughly 1050–1200 cm^−1^, primarily associated with the appearance of C-O groups as well as ozonide and peroxide functional groups [37]. These can form new bonds between the phases, thereby improving the mechanical properties of the prepared vulcanizates. In the spectra of GTRs treated in an inert atmosphere, these oxygen-containing groups are not visible. In this case, the spectrum of the treated GTR closely matched the reference, so the difference spectrum in this case, is practically a straight line. Our assumption has thus been confirmed, as the surface of the GTR was activated during irradiation in water, while in a nitrogen atmosphere, new oxygen-containing functional groups did not form on the surface due to the absence of oxygen.

**Fig. 5:**
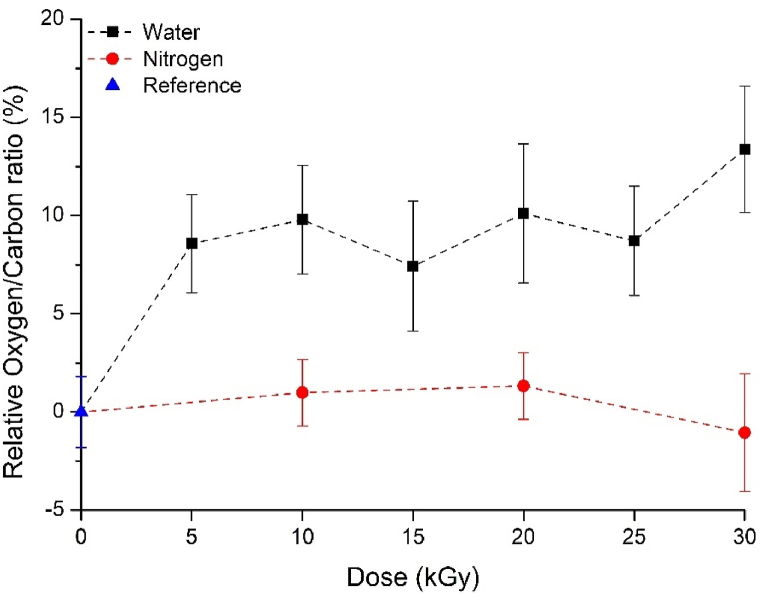
The relative O/C ratio of the examined GTR samples.

In order to quantitatively analyze the oxygen-containing groups appearing on the surface due to the radiation treatment, we performed XPS. In Fig. 5, we summarized the relative oxygen/carbon ratios calculated from XPS. In the evaluation, we used the reference O/C ratio as a basis and examined the deviation from it for the other samples. The results indicate that the amount of oxygen on the surface significantly increased by approximately ∼10 % due to irradiation in water. It also did not depend on the absorbed dose in the examined range (<30 kGy). In the case of GTR treated in an inert atmosphere, the oxygen/carbon ratio did not change compared to the reference, and the absorbed dose had no effect on it. Therefore, it can be concluded that oxygen-containing groups appear on the surface during irradiation in water, as evidenced by the increase in the O/C ratio (∼10 %). In a nitrogen atmosphere, the amount of oxygen on the surface did not increase compared to the reference, indicating that surface activation did not occur due to the absence of oxygen.

**Fig. 6:**
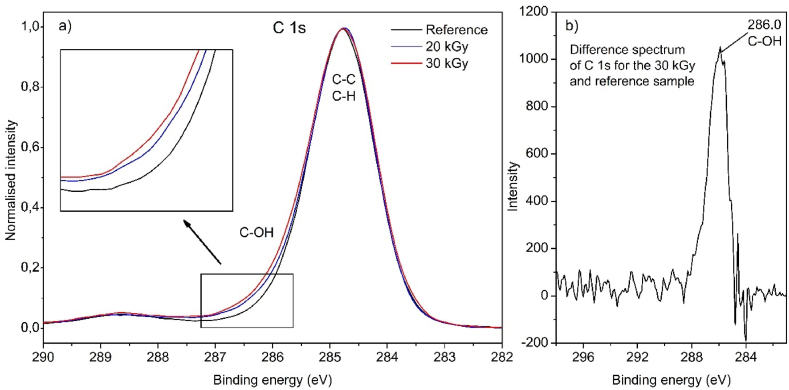
The a) C 1s spectra of the reference and irradiated GTRs in water b) difference spectrum of C 1s for the 30 kGy (irradiated in water) and the reference sample.

In Fig. 6a, we showed the C 1s spectrum of GTRs irradiated in water with absorbed doses of 20 kGy and 30 kGy because these samples exhibited the highest amount of surface oxygen. Fig. 6b shows the difference spectrum between the 30 kGy and the reference (untreated) sample. The results indicate that surface oxygen is primarily associated with C-OH groups [38], as proved by the difference spectrum. It correlates well with the FTIR results. Unfortunately, other functional groups detected by FTIR are not discernible with XPS.

### 3.2 Properties of the vulcanizates containing GTR

We summarized the vulcanization properties of the mixtures containing GTR in Table 4. The untreated reference is annotated as GTR, while the samples containing irradiated GTR are abbreviated as GTR/X-Y, where X stands for the applied atmosphere (W for water and N for nitrogen), where Y stands for the absorbed dose. Compared to the reference, both treatments increased the maximum torque (S'_max_). However, in the latter case, this increase is of a smaller extent, possibly related to the cross-linking of the GTR. However, as a result of irradiation in water, the maximum torque increased significantly, by more than 25 % in the case of 30 kGy. This indicates that the cross-link density of the vulcanizates increased due to the surface treatment. A difference is noticeable in both the vulcanization times (t_90_) and the speed of vulcanization (peak rate). In both the reference and nitrogen cases, The t_90_ times of the mixtures containing GTR treated in water are the lowest, while the peak rates are higher. The results indicate that during surface treatment (water irradiation), the active groups appearing on the surface participated in the vulcanization process, accelerating it. This reduced the vulcanization time and increased the maximum torque, thereby increasing the quantity of cross-links that formed.

**Table 4:** The vulcanization properties of the investigated samples containing GTR.

Sample	S'_max_ (dNm)	t_90_ (min)	Peak rate (dNm/min)
GTR	14.4	3.2	16.9
GTR/W-5	17.5	2.8	18.9
GTR/W-10	16.8	2.7	18.4
GTR/W-15	17.5	2.7	19.4
GTR/W-20	17.6	2.8	19.3
GTR/W-25	17.9	2.8	20.0
GTR/W-30	18.1	2.8	19.7
GTR/N-5	16.3	3.5	15.3
GTR/N-10	16.7	3.3	16.9
GTR/N-15	17.4	3.2	17.9
GTR/N-20	16.5	3.3	17.1
GTR/N-25	15.7	3.2	15.4
GTR/N-30	14.7	3.4	13.3

**Fig. 7:**
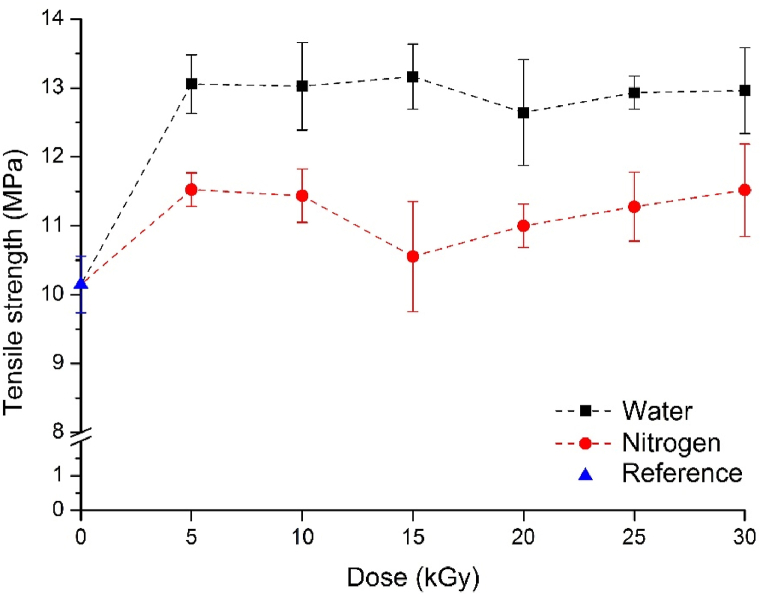
The tensile strength of the vulcanizates containing GTR.

The materials containing radiation-treated GTR in a nitrogen atmosphere had higher tensile strength than the untreated reference (Fig. 7). However, this increase is not as significant as in the case of vulcanizates containing surface-activated GTR. The reason for the increase is likely that active sites remained on the GTR after the radiation treatment in the inert atmosphere, as they could not react in the nitrogen atmosphere. These sites may have reacted with the oxygen in the air or with the matrix itself, thereby forming a better connection between the phases. However, it is noticeable that at higher doses, tensile strength decreases and does not approach that of the vulcanizates containing GTR irradiated in water. In this case, due to the lack of surface activation, a strong connection was not established between the phases.

The tensile strength of vulcanizates containing GTR that was activated in water increased significantly, by approximately 30 %, even at the low dose of 5 kGy. This increment was maintained even at higher doses. The GTR and natural rubber matrix established a better connection due to surface activation. The active groups that formed (peroxides, hydroxyl groups, etc.) created new bonds between the GTR and the matrix, resulting in a substantial increase in tensile strength. Economically, it is particularly advantageous that a significant improvement in strength was achieved even with a low dose of 5 kGy.

**Fig. 8:**
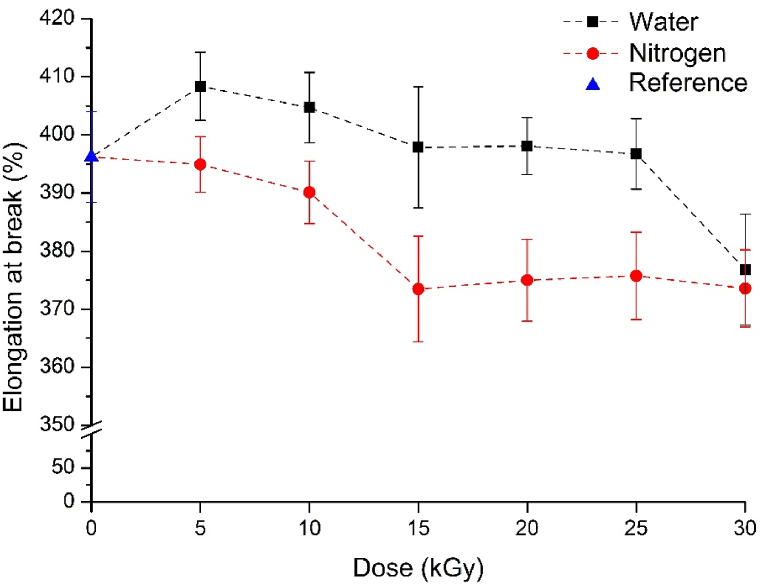
Elongation at break of the vulcanizates containing GTR.

The elongation at break of the examined vulcanizates are shown in Fig. 8. In the case of materials containing GTR treated under a nitrogen atmosphere, the elongation at break decreased as the dose was increased to 15 kGy. Above this dose, it stabilized at an almost constant value, which was lower than that of the reference. The elongation at break of vulcanizates containing surface-activated GTR increased slightly compared to the reference, and only at the highest dose did it decrease below the reference. In this case, the improved connection between phases resulted in outstanding elongation at break (∼400 %) for the natural rubber–based filled systems, while in the nitrogen atmosphere, due to the absence of surface-active groups, adhesion did not improve, and consequently, elongation at break did not show improvement. Therefore, the strength of the vulcanizates increased when irradiated in water, without compromising elongation at break, which is an exceptional result for subsequent utilization.

**Fig. 9:**
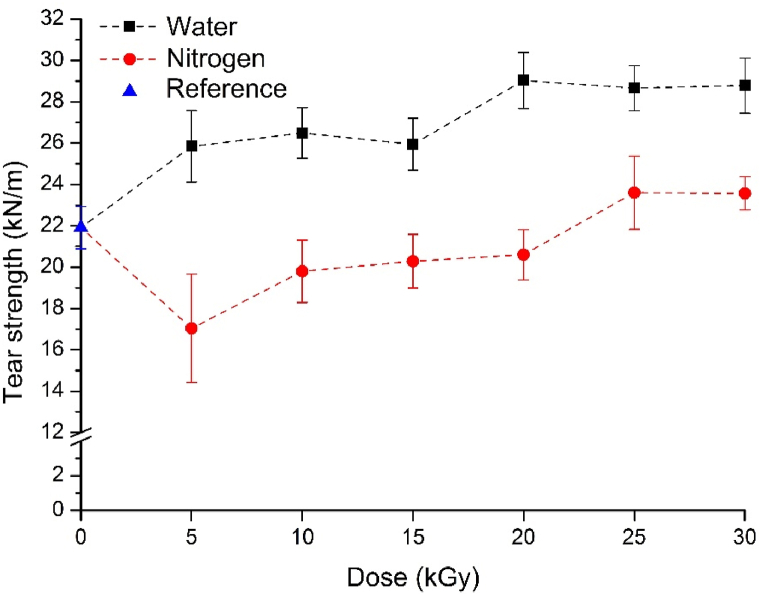
Tear strength of the vulcanizates containing GTR.

In the case of vulcanizates containing GTR treated in an inert atmosphere, tear strength decreased compared to the reference, with only 10, 25, and 30 kGy yielding results close to the reference (Fig. 9). The reason for this is that surface activation did not occur due to the treatment in an oxygen-free environment, preventing a better connection between the matrix and the ground tire rubber. Consequently, tear strength did not improve.

In contrast, the water radiolysis treatment increased the tear strength of vulcanizates containing surface-activated GTR. Even at 5 kGy, the improvement was 18 %, while the best result was obtained at 20 kGy, where tear strength increased by more than 32 % compared to the reference. In the case of tear strength, it is crucial for the filler and matrix to work well together, and it is evident that the water treatment produces a better connection between the phases through surface activation.

**Fig. 10:**
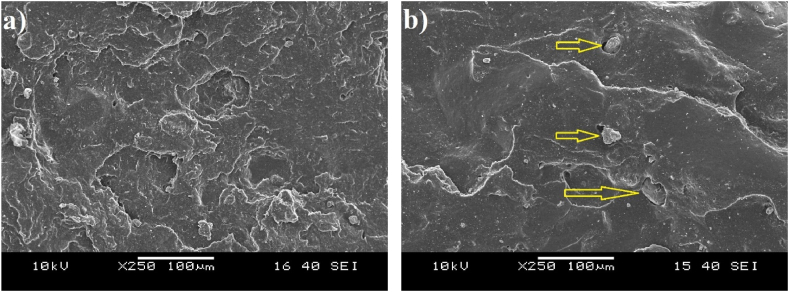
The morphology of the vulcanizates containing a) GTR irradiated with 20 kGy in water; b) GTR irradiated with 20 kGy in a nitrogen atmosphere (tear strength specimens).

Fig. 10 shows the fracture surfaces of the tear test specimens. Fig. 10a shows the fracture surface of the vulcanizate containing GTR irradiated with 20 kGy in water. It has a segmented morphology, which is indicative of tougher failure behavior. In addition, free, protruding GTR particles are not visible on microscopes, and even GTR particles cannot be distinguished from the surrounding matrix.In this case, due to the excellent interphase adhesion, crack propagation is impeded when the crack reaches a GTR particle. The crack most likely propagated in the GTR–matrix interphase, but due to the excellent interphase bonding between the phases, it leads to a more resilient failure, resulting in a more segmented fracture surface and higher tear strength.

In contrast, Fig. 10b depicts the fracture surface of the vulcanizate containing GTR irradiated with 20 kGy in an inert atmosphere. The surface is much smoother, less segmented, and in several places, free GTR particles are visible. These particles have pulled away from the matrix due to inadequate bonding during failure. In this case, when the crack reached a GTR particle, it stopped, too. However, it easily continued along the GTR–matrix interface. As a result of this, free GTR particles are visible, and the smooth surface indicates that the failure was less tough in this case.

Smaller particles can be seen in both images. It is not clear from these images whether these are GTR particles or whether they come from the surrounding material but we can conclude that they do not play a determining role in the failure.

## 4 Conclusions

In this study, we successfully applied a water-based, low-dose ionizing radiation treatment to activate the surface of ground tire rubber, thus improving compatibility in mixtures containing GTR. To further investigate the phenomena occurring on the surface, we also conducted radiation treatments in an inert (nitrogen) atmosphere. Irradiation in water resulted in a decrease in cross-link density (∼10 %), while in an inert atmosphere, it increased (∼10 %), with minimal degradation (1–2 %) in both cases. The strong oxidizing agents that appeared because of the radiolysis of water introduced new functional groups on the surface (hydroxyl, carbonyl, peroxide, etc.), which we identified using FTIR spectroscopy. Surface oxygen was quantified through XPS analysis, revealing an approximately 10 % increase in surface oxygen concentration compared to the reference, while there was no change in surface properties in the case of treatment in an inert atmosphere.

We examined the vulcanization properties and determined that functional groups appearing during surface activation are involved in vulcanization. This is evidenced by an increase in the vulcanization rate (peak rate), which results in a reduction in vulcanization time compared to the reference and mixtures containing GTR treated in an inert atmosphere. Maximum torque also increased, correlating with the increased cross-link density, as these groups facilitate the formation of new bonds between phases. The improvement in compatibility is shown in the mechanical properties, as the tensile strength of vulcanizates containing surface-activated GTR increased by 30 %, while elongation at break remained high (∼400 %) and, in some cases, even improved. Interfacial adhesion is characterized well by tear strength, which increased by 32 % as a result of the radiation treatment in water. In samples treated in a nitrogen atmosphere, such a significant improvement was not observed, which can be attributed to the absence of surface activation. The fracture surfaces of the tear-tested specimens indicate that adhesion between the phases was excellent due to the irradiation water, which resulted in a segmented fracture surface, indicating toughness. In contrast, in the vulcanizates containing GTR irradiated in a nitrogen atmosphere, the fracture surfaces were smooth with visible GTR particles, due to inadequate compatibility between the phases.

In conclusion, we have demonstrated that oxygen-containing active functional groups appear on the surface of the GTR as a result of radiolysis in water, capable of establishing a better connection between the GTR and the matrix, thus improving the mechanical properties of vulcanizates. By applying a nitrogen atmosphere, we were able to separate the effects of surface activation and structural changes of the GTR on the properties of vulcanizates. In the case of the low doses applied, there were slight changes in the structure caused by the treatment in either nitrogen or water. However, in the inert atmosphere, there was no surface activation and so the mechanical properties of the vulcanizates did not improve. Using this technology, the application range of these materials can be expanded, and the amount of rubber waste in the environment can be reduced. It is also noteworthy that even at very low doses, such as 5 kGy, remarkable results were achieved, which is advantageous from both environmental and industrial feasibility perspectives.

## Data availability statement

Data will be made available on request.

## Ethics statement

Review and/or approval by an ethics committee was not needed for this study because no human or animal participation was involved.

## CRediT authorship contribution statement

**Lóránt Kiss:** Writing – original draft, Visualization, Validation, Investigation, Formal analysis. **Alexandra Erzsébet Berényi:** Validation, Investigation. **Miklós Németh:** Writing – original draft, Visualization, Validation, Investigation. **Anna Tegze:** Investigation, Formal analysis. **Renáta Homlok:** Validation, Investigation, Conceptualization. **Erzsébet Takács:** Writing – review & editing, Supervision, Methodology, Conceptualization. **László Mészáros:** Writing – review & editing, Writing – original draft, Supervision, Methodology, Funding acquisition, Conceptualization.

## Declaration of competing interest

The authors declare that they have no known competing financial interests or personal relationships that could have appeared to influence the work reported in this paper.

## References

[1] Fazli A., Rodrigue D. (2020). Recycling waste tires into ground tire rubber (GTR)/rubber compounds: a review. J. Compos. Sci..

[2] Boon Teo, Ang (2023). Synthesis and evaluation of poly(isoprene-co-acrylonitrile) as synthetic rubber with enhanced oil resistance. Express Polym. Lett..

[3] Karger-Kocsis J., Meszaros L., Barany T. (2013). Ground tyre rubber (GTR) in thermoplastics, thermosets, and rubbers. J. Mater. Sci..

[4] Adhikari J., Das A., Sinha T., Saha P., Kim J.K., Kim J.K., Saha P., Thomas S., Haponiuk J.T., Aswathi M.K. (2018). Rubber Recycling: Challenges and Developments.

[5] Susik A., Rodak A., Cañavate J., Colom X., Wang S., Formela K. (2023). Processing, mechanical and morphological properties of GTR modified by SBS copolymers. Mater.

[6] Formela K., Eyigöz B. (2024). Planetary roller extruders in the sustainable development of polymer blends and composites – past, present and future. Express Polym. Lett..

[7] Navarro F.J., Partal P., Martınez-Boza F., Valencia C., Gallegos C. (2002). Rheological characteristics of ground tire rubber-modified bitumens. Chem. Eng. J..

[8] Formela K., Wąsowicz D., Formela M., Hejna A., Haponiuk J. (2015). Curing characteristics, mechanical and thermal properties of reclaimed ground tire rubber cured with various vulcanizing systems. Iran. Polym. J. (Engl. Ed.).

[9] Nicolas C., Rachel L., Maria Lluisa M. (2023). A comparison of the mechanical behaviour of natural rubber-based blends using waste rubber particles obtained by cryogrinding and high-shear mixing. Express Polym. Lett..

[10] Shaker R., Rodrigue D. (2019). Rotomolding of thermoplastic elastomers based on low-density polyethylene and recycled natural rubber. Appl. Sci..

[11] Liu S., Peng Z., Zhang Y., Rodrigue D., Wang S. (2022). Compatibilized thermoplastic elastomers based on highly filled polyethylene with ground tire rubber. J. Appl. Polym. Sci..

[12] Formela K. (2022). Waste tire rubber-based materials: processing, performance properties and development strategies. Adv. Ind. Eng. Polym. Res..

[13] Rodak A., Susik A., Kowalkowska-Zedler D., Zedler Ł., Formela K. (2023). Cross-linking, morphology, and physico-mechanical properties of GTR/SBS blends: dicumyl peroxide vs. sulfur system. Mater.

[14] Simon D.Á., Bárány T. (2023). Microwave devulcanization of ground tire rubber and its improved utilization in natural rubber compounds. ACS Sustainable Chem. Eng..

[15] Seghar S., Asaro L., Rolland-Monnet M., Aït Hocine N. (2019). Thermo-mechanical devulcanization and recycling of rubber industry waste. Resour. Conserv. Recycl..

[16] Asaro L., Gratton M., Poirot N., Seghar S., Aït Hocine N. (2020). Devulcanization of natural rubber industry waste in supercritical carbon dioxide combined with diphenyl disulfide. Waste Manage. (Tucson, Ariz.).

[17] Cheng Y., Wei Y., Wu H., Zhang T., Li S., Zhu N., Zhang Q., Li W. (2024). Biodegradation of vulcanized natural rubber by enriched bacterial consortia. Chem. Eng. J..

[18] Zhang X., Lu C., Liang M. (2009). Properties of natural rubber vulcanizates containing mechanochemically devulcanized ground tire rubber. J. Polym. Res..

[19] Phiri M.M., Phiri M.J., Formela K., Hlangothi S.P. (2021). Chemical surface etching methods for ground tire rubber as sustainable approach for environmentally-friendly composites development-a review. Compos. Part B Eng..

[20] Hejna A., Olszewski A., Zedler Ł., Kosmela P., Formela K. (2021). The impact of ground tire rubber oxidation with H_2_O_2_ and KMnO_4_ on the structure and performance of flexible polyurethane/ground tire rubber composite foams. Mater.

[21] Sonnier R., Leroy E., Clerc L., Bergeret A., Lopez-Cuesta J.M., Bretelle A.S., Ienny P. (2008). Compatibilizing thermoplastic/ground tyre rubber powder blends: efficiency and limits. Polym. Test..

[22] Sonnier R., Leroy E., Clerc L., Bergeret A., Lopez-Cuesta J.M. (2007). Polyethylene/ground tyre rubber blends: influence of particle morphology and oxidation on mechanical properties. Polym. Test..

[23] Sonnier R., Leroy E., Clerc L., Bergeret A., Lopez-Cuesta J.M. (2006). Compatibilisation of polyethylene/ground tyre rubber blends by γ irradiation. Polym. Degrad. Stabil..

[24] Khusyainova D.N., Shapagin A.V., Ponomarev A.V. (2022). Radiation-stimulated oxidation of the plastic surface in a water-air flow. Radiat. Phys. Chem..

[25] Cao X.-W., Luo J., Cao Y., Yin X.-C., He G.-J., Peng X.-F., Xu B.-P. (2014). Structure and properties of deeply oxidized waster rubber crumb through long time ozonization. Polym. Degrad. Stabil..

[26] He L., Ma Y., Liu Q., Mu Y. (2016). Surface modification of crumb rubber and its influence on the mechanical properties of rubber-cement concrete. Constr. Build. Mater..

[27] Ponomarev A.V., Gohs U., T Ratnam C., Horak C. (2022). Keystone and stumbling blocks in the use of ionizing radiation for recycling plastics. Radiat. Phys. Chem..

[28] Mészáros L., Bárány T., Czvikovszky T. (2012). EB-promoted recycling of waste tire rubber with polyolefins. Radiat. Phys. Chem..

[29] Mészáros L., Fejős M., Bárány T. (2012). Mechanical properties of recycled LDPE/EVA/ground tyre rubber blends: effects of EVA content and postirradiation. J. Appl. Polym. Sci..

[30] Zaharescu T. (2023). Synergistic effect of silica nanoparticles assisted by rosemary powder in the stabilization of styrene-isoprene-styrene triblock copolymer. Radiat. Phys. Chem..

[31] Ratnam C., Ramarad S., Khalid M., Noraini N. (2013). Effect of pre-irradiation of waste tire dust on the properties of ethylene vinyl acetate/waste tire dust blend (EVA/WTD) blends. J. Compos. Biodegrad. Polym..

[32] Kiss L., Simon D.Á., Bárány T., Mészáros L. (2022). Synergistic effects of gamma pre-irradiation and additional vulcanizing agent in case of ground tire rubber containing vulcanizates. Radiat. Phys. Chem..

[33] Kiss L., Mészáros L. (2024). Recycling waste tire rubber through an innovative water-medium ionizing radiation treatment: enhancing compatibility and mechanical performance. Radiat. Phys. Chem..

[34] Flory P.J., Rehner J.J. (1943). Statistical mechanics of cross-linked polymer networks II. swelling. J. Chem. Phys..

[35] Ellis B., Welding G.N. (1964). Estimation, from swelling, of the structural contribution of chemical reactions to the vulcanization of natural rubber. Part II. Estimation of equilibrium degree of swelling. Rubber Chem. Technol..

[36] Nandiyanto A.B.D., Oktiani R., Ragadhita R. (2019). How to read and interpret FTIR spectroscope of organic material. Indones. J. Sci. Technol..

[37] Cataldo F., Ursini O., Angelini G. (2010). Surface oxidation of rubber crumb with ozone. Polym. Degrad. Stabil..

[38] Biesinger M.C. (2022). Accessing the robustness of adventitious carbon for charge referencing (correction) purposes in XPS analysis: insights from a multi-user facility data review. Appl. Surf. Sci..

